# Functional Interaction of Nuclear Domain 10 and Its Components with Cytomegalovirus after Infections: Cross-Species Host Cells versus Native Cells

**DOI:** 10.1371/journal.pone.0019187

**Published:** 2011-04-28

**Authors:** Ruth Cruz Cosme, Francisco Puerta Martínez, Qiyi Tang

**Affiliations:** Department of Microbiology/AIDS Research Program, Ponce School of Medicine and Health Sciences, Ponce, Puerto Rico, United States of America; Karolinska Institutet, Sweden

## Abstract

Species-specificity is one of the major characteristics of cytomegaloviruses (CMVs) and is the primary reason for the lack of a mouse model for the direct infection of human CMV (HCMV). It has been determined that CMV cross-species infections are blocked at the post-entry level by intrinsic cellular defense mechanisms, but few details are known. It is important to explore how CMVs interact with the subnuclear structure of the cross-species host cell. In our present study, we discovered that nuclear domain 10 (ND10) of human cells was not disrupted by murine CMV (MCMV) and that the ND10 of mouse cells was not disrupted by HCMV, although the ND10-disrupting protein, immediate-early protein 1 (IE1), also colocalized with ND10 in cross-species infections. In addition, we found that the UL131-repaired HCMV strain AD169 (vDW215-BADrUL131) can infect mouse cells to produce immediate-early (IE) and early (E) proteins but that neither DNA replication nor viral particles were detectable in mouse cells. Unrepaired AD169 can express IE1 only in mouse cells. In both HCMV-infected mouse cells and MCMV-infected human cells, the knocking-down of ND10 components (PML, Daxx, and SP100) resulted in significantly increased viral-protein production. Our observations provide evidence to support our hypothesis that ND10 and ND10 components might be important defensive factors against the CMV cross-species infection.

## Introduction

Cytomegaloviruses (CMVs) are a β-subfamily of herpes viruses. Many types of cells (including fibroblast, epithelial, endothelial, and hematopoietic cells) are permissive for CMV infection, which infection results in the production of infectious particles [1], but CMV infection and replication are limited to a narrow host range [2], [3]. For example, murine CMV (MCMV) can produce viral particles in both mouse and rat cells, while rat CMV (RCMV) cannot successfully replicate in mouse cells [4], [5]. Similar observations were also reported for human CMV (HCMV) and simian CMV (SCMV). SCMV productively infected human and monkey cells, but HCMV failed to replicate in monkey cells [3]. CMV replication in native host cells is a well-defined sequential process: entry into cells, immediate-early (IE) and early (E) gene expression, DNA replication, late gene expression, and viral production [6]. Blocking any stage will cause the failure of infection. It has been determined that both CMV cross-species infections and low MOI (multiplicity of infection) infections in permissive cells are blocked at the post-entry level by intrinsic cellular defense mechanisms [3], [6], but few details are known.

We and others recently discovered that viruses encode gene products that counter cellular defenses in human cells, which preventive action can help MCMV to successfully infect human cells [7], [8]. For instance, we discovered that intrinsic cellular defense mechanisms are involved in blocking MCMV infection in human cells and that these mechanisms can be overcome by HCMV-encoded proteins (such as immediate-early protein 1—IE1), resulting in successful cross-species infection [7]. The Brune group discovered that the inhibition of apoptosis by the overexpression of Bcl-2 and other apoptosis inhibitors caused the successful replication of MCMV in human cells [8]. However, very few efforts have attempted to determine how HCMV replication is blocked in mouse cells other than to observe that HCMV infection in mouse cells is blocked at the IE stage [3]. The significance of successfully infecting mouse cells with HCMV is that doing so would enable the development of an HCMV mouse model. We are also curious whether any nuclear structure (and its components) is involved in blocking cytomegalovirus cross-species infection.

A nuclear structure called ND10 (nuclear domain 10) has been attracting intense attention from virologists due to the functional interaction of its components with viruses. Several herpes viruses (e.g., Herpes simplex virus type-1 [HSV-1], cytomegalovirus [CMV], and Epstein-bar virus [EBV]) were found to be capable of disrupting ND10 [9], [10], [11], and various viral proteins have been identified as being related to ND10 and ND10 proteins, which identification has been summarized by Dr. Kalejta and colleagues [12]. Recently, accumulated evidence showed that major ND10 components (PML, Daxx, and SP100) have negative impacts on the herpesviruses [13], [14], [15], [16], [17], [18], [19], [20], [21], [22], [23], [24]. Therefore, it has been assumed that ND10 defends against herpes viral infection, but this assumption is contradicted by the fact that several DNA viruses replicate DNA and transcribe RNA predominately at ND10 [25], [26].

More recently, the Brune group isolated a naturally acquired mutant MCMV that was able to replicate rapidly and to high titers in human retinal pigment epithelial (RPE-1) cells [27]. The interesting observation that the ability of mutated MCMV to disrupt ND10 seems to be related to viral production [27] initiated our investigation on whether the disruption of ND10 might be related to HCMV infection in mouse cells. In the present study, we discovered that HCMV infection in mouse cells can express IE and many early genes and is blocked before DNA replication. In addition, we show that ND10 colocalizes with IE1 in cross-species infections but is not dispersed by CMV in such infections (HCMV in mouse cells and MCMV in human cells) and that ND10 components are involved in blocking viral gene expression in both MCMV and HCMV cross-species infections.

## Materials and Methods

### Cells and viruses

The following cell lines were used: NIH3T3 (ATCC), Mrc-5 cells (ATCC), and Ad5 E1A-transformed human epithelial kidney cell 293 (HEK293, ATCC). Cells were maintained in Dulbecco's modified Eagle's medium (DMEM) supplemented with 10% fetal calf serum (FCS) and 1% penicillin-streptomycin (PS). The MCMV Smith strain was from ATCC. The HCMV Towne and AD169 strains (ATCC) were kept in our laboratory; the clinical strains of HCMV included FIX [VIR1814, provided by Dr. Gerna in Italy [28]] and Toledo [provided by Dr. Zhu in UMDNJ [29]]; the UL131-repaired HCMV AD169 strain (vDW215-BADrUL131) was provided by Dr. Shenk (Princeton University) [29].

### RNA interference

In order to knock down the expression of the ND10 components, we transfected plasmids, thereby producing small interference RNA (siRNA) against each component. All the siRNA-producing plasmids were bought from Santa Cruz Biotechnology, Inc. (Santa Cruz, CA). The shRNA plasmids are shown in Table 1. An shRNA plasmid (sc-108060) encoding of a scrambled shRNA sequence that does not lead to the specific degradation of any cellular message is used as control. For Western blot analysis, 100 pmol of siRNA plasmid was used for each well of a 24-well plate of HEK293 cells or NIH3T3 cells; 24 h after transfection, cells were super-infected with MCMVIE1/3gfp or the UL131-repaired AD169 (vDW215-BADrUL131) at an MOI of 1 for another 24 hours, then the cells were harvested and lysed for protein detection.

**Table 1:** shRNA plasmids used in this study.

Mouse cell	Human cell
SP-100 (sc-41033-SH)	SP-100 (sc-41032-SH)
PML (sc-36283-SH)	PML (sc-36284-SH);
Daxx (sc-35177-SH)	Daxx (sc-35178-SH)

### Antibodies

The human and mouse ND10-associated proteins were recognized using the following antibodies from Santa Cruz Biotechnology, Inc. (Santa Cruz, CA): Monoclonal antibody (mAb) (PG-M3, sc-966) against human PML, polyclonal antibody (H-238, sc-5621) against human and mouse PML, polyclonal antibody against human and mouse Daxx (M-112, sc-7152), and polyclonal antibody against human SP100 (H-60, sc-25568) and mouse SP100 (M-75, sc-25569). MAb against tubulin came from Sigma Co. (St. Louis, MO). MAb against MCMV IE1 and M112/113 (E1) were generously provided by S. Jonjic (Croatia). Monoclonal antibody against HCMV IE1, mAb810, came from Millipore (Billerica, MA).

### Detection of Viral DNA replication by PCR

Cells (NIH3T3 or Mrc-5) were infected with HCMV AD169 (vDW215-BADrUL131) at an MOI of 1. Viral DNA was isolated using the Hirt method [30]. PCR was performed on HCMV DNA using the following primers: forward-5′ GTC AAA CAG ATT AAG GTT CGA GTG-3′ and reverse-5′ TTA CTG GTC AGC CTT GCT TCT AGT 3′.

### Immunohistochemistry

The localization of ND10 and viral proteins by immunohistochemistry has been described [31]. Briefly, cells were seeded on coverslips and washed twice with phosphate-buffered saline (PBS) 24 h later, fixed in 1% paraformaldehyde for 10 min at room temperature, again washed twice with PBS, and permeabilized with 0.2% Triton X-100 on ice for 20 min. Primary antibody was added and incubated for 30 min at room temperature. Cells were then washed twice with PBS. Secondary antibody (labeled with Texas Red or fluorescein isothiocyanate [green] of either anti-rabbit or anti-mouse IgG) was added and incubated for an additional 30 min at room temperature. After a final wash with PBS, cells were stained with Hoechst 33258.

### Immunoblot analysis

Proteins were separated by SDS-7.5% polyacrylamide gel electrophoresis, transferred to nitrocellulose membranes, and probed according to standard procedures. Membranes were stripped with stripping buffer (100 mM ß-mercaptoethanol, 2% SDS, 62.5 mM Tris-HCl, pH 6.8), washed with PBS-0.1% Tween 20, and used to detect additional proteins.

### Confocal microscopy

Cells were examined at 100× magnification with a Leica TCS SPII confocal laser scanning system equipped with a water-cooled argon-krypton laser. Two wavelength channels (495 and 590 nm) were recorded simultaneously or sequentially. Power and integration were adjusted to minimize bleed-through between the green and far red channels prior to data acquisition. Digital images obtained were cropped and adjusted for contrast with Photoshop.

## Results

### ND10 were not dispersed by cytomegalovirus cross-species infection but co-localized with the ND10-disrupting protein (IE1)

**Figure 1:**
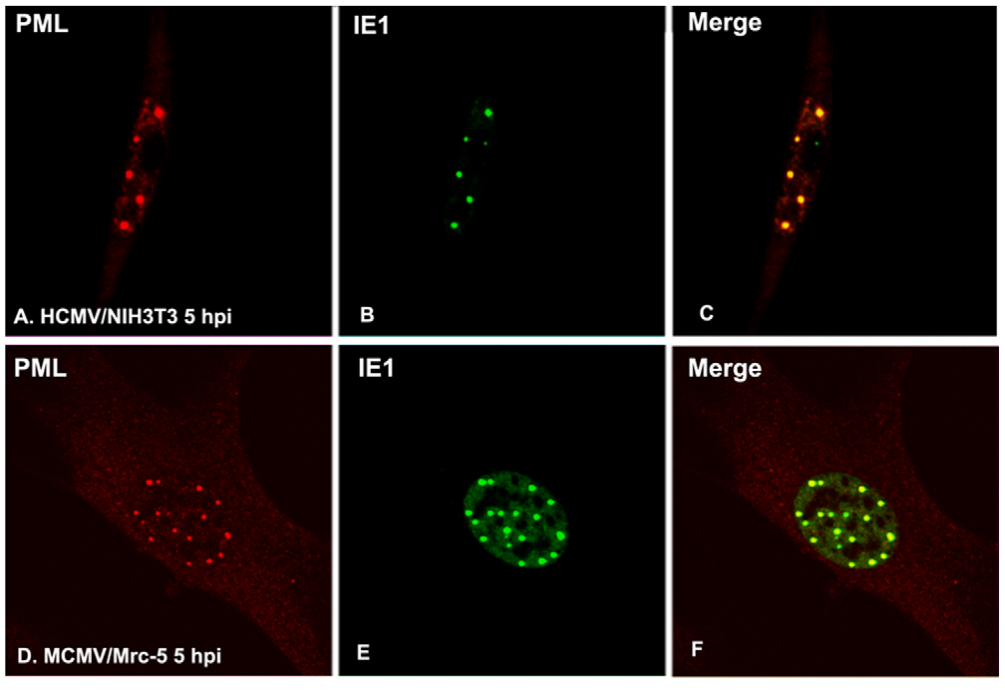
Immunofluorescent assay to show IE1 and ND10 in CMV cross-species infection. **A–C**: After HCMV infection in NIH3T3 cells for 5 hour, cells were stained with anti-PML antibody (rabbit) to show ND10 (in red) (A); anti-IE1 antibody (mouse) was used to show IE1 (in green) (B); the merged picture is shown in C. **D–F**: After MCMV infection in Mrc-5 cells for 5 hour, cells were stained with anti-PML antibody (Rabbit) to show ND10 (in red) (D); anti-IE1 antibody (mouse) was used to show IE1 (in green) (E); the merged picture is shown in F.

At least two aspects of the relationship between ND10 and DNA viruses have been described: several DNA virus-encoded proteins colocalize with ND10, and some of them can disperse ND10 [25], [32]. The fact that CMV IE1 disrupts the ND10 of native host cells supports the speculation that ND10 acts in a defensive capacity. We wondered whether the IE1 of cytomegalovirus could behave differently in cross-species infections. To determine this, we performed an immunofluorescence assay to verify whether CMV IE1 still colocalizes with ND10, such as occurs in the infection of native host cells [10], [26]. As shown in Fig. 1, we stained HCMV IE1 (green, B and E) and ND10 (anti-PML in red, A and D) at five hours post-infection (hpi) of the HCMV UL131-repaired AD169 (vDW215-BADrUL131) in NIH3T3 cells (A–C) and of the wt (wild type) MCMV in human cells (D–F). Clearly, at the very early stage, IE1 presented as patterns of speckles. The IE1 speckles colocalized with ND10 after CMV cross-species infection in host cells. Therefore, the distribution of IE1 at the very early stage of infection was not different in cross-species infection compared with native infection (e.g., HCMV in human cells and MCMV in mouse cells).

**Figure 2:**
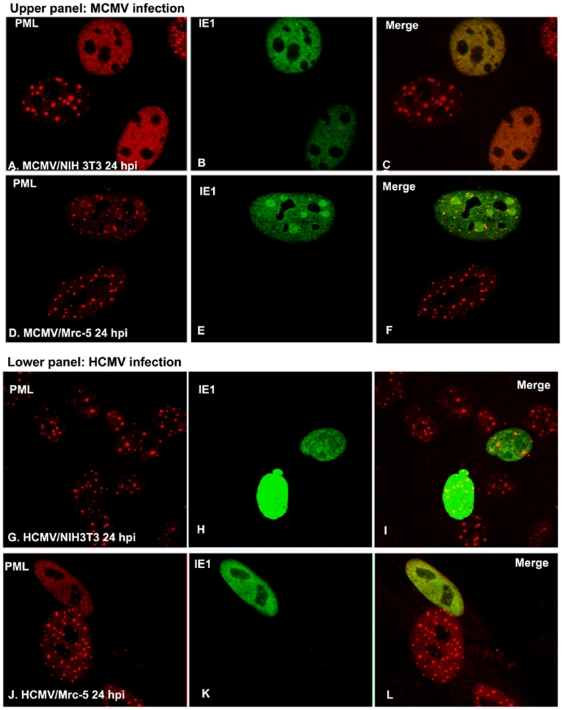
Immunofluorescent assay to show cytomegalovirus infection and ND10. **A–C**: After MCMV infection in NIH3T3 cells for 24 hours, cells were stained with anti-PML antibody (rabbit) to show ND10 (in red) (A); anti-IE1 antibody (mouse) was used to show IE1 (in green) (B); the merged picture is shown in C. **D–F**: After MCMV infection in Mrc-5 cells for 24 hours, cells were stained with anti-PML antibody (rabbit) to show ND10 (in red) (D); anti-IE1 antibody (mouse) was used to show IE1 (in green) (E); the merged picture is shown in F. **G–H**: After HCMV infection in NIH3T3 cells for 24 hours, cells were stained with anti-PML antibody (rabbit) to show ND10 (in red) (G); anti-IE1 antibody (mouse) was used to show IE1 (in green) (H); the merged picture was shown in I. **J–L**: After HCMV infection in Mrc-5 cells for 24 hours, cells were stained with anti-PML antibody (rabbit) to show ND10 (in red) (J); anti-IE1 antibody (mouse) was used to show IE1 (in green) (K); the merged picture is shown in L.

Next, we performed another IFA to analyze the ability of IE1 to disperse ND10 in cross-species–infected cells. We infected wt MCMV into Mrc-5 cells and HCMV into NIH3T3 cells for 24 hours. Cells were fixed and permeabilized and stained with anti-PML to show ND10 (red, Fig. 2A, D, G, and J) and anti-IE1 to show the distribution of IE1 (green, Fig. 2B, E, H, and K). As can be seen in HCMV-infected human cells and MCMV-infected mouse cells, IE1 was diffusely distributed in the nucleus at 24 hpi. Interestingly, the IE1 of MCMV formed domains (Fig. 2 E) in human cells and lost the ability to disperse ND10, their distribution being different from that found in MCMV-infected mouse cells (Fig. 2 A–C). Similarly, HCMV IE1 also lost the ability to disperse ND10 in mouse cells (Fig. 2 G–I), which was controlled by an IFA of the HCMV-infected Mrc-5 cells in which the ND10 was dispersed (Fig. 2 J–L). However, HCMV IE1 did not form domains in NIH3T3 cells but diffused. The differential distribution of IE1 and the inability to disperse ND10 in cross-infection might be related to the failure to induce a productive cytomegalovirus cross-species infection.

**Figure 3:**
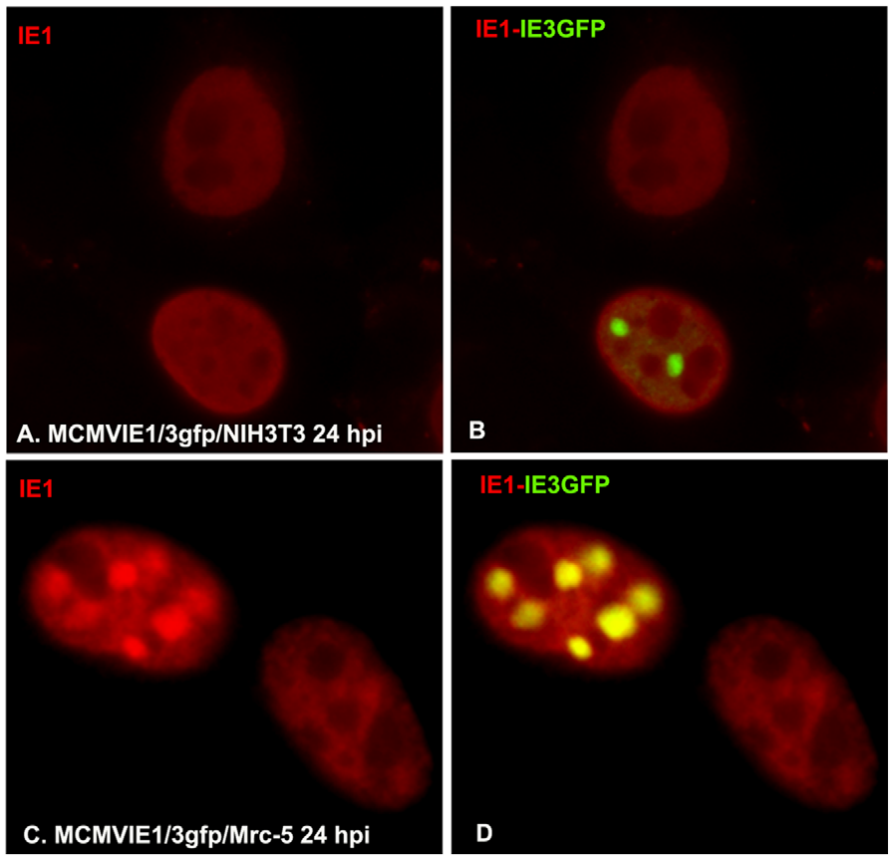
Immunofluorescent assay to show IE1 and IE3 in murine cytomegalovirus-infected human and mouse cells. Upper: MCMVIE1/3gfp-infected NIH3T3 cells to show distribution of IE1 (left) and IE1_IE3 (right). Lower: MCMVIE1/3gfp-infected Mrc-5 cells to show distribution of IE1 (left) and IE1_IE3 (right).

Previously, we discovered that MCMV IE1 and IE3 have no associations with each other in infected mouse cells [33]. We wanted to know whether IE1 could be related to IE3 in MCMV-infected human cells. For the detection of IE3, we infected NIH3T3 cells with MCMVIE1/3gfp in which IE1 was kept intact and EGFP was also fused to the C-terminus of IE3 [33]. At 24 hpi, we performed IFA to stain IE1 red (Fig. 3 A and C) and IE3 green (Fig. 3B and D). As can be seen, in MCMV1/3gfp-infected mouse cells, IE1 was shown not to be related to IE3 (Fig. 3A and B), which is consistent with our previous report [33]. However, IE1 formed domains that colocalized with IE3 domains in MCMV-infected human cells (Fig. 3C and D). The biological impact of the association between IE1 and IE3 in MCMV-infected human cells on viral infection still remains unclear.

### Detection of HCMV proteins in infected NIH3T3 cells

Two decades ago, Lafemina and Hayward reported that HCMV can enter mouse cells and produce only IE1, its infection being blocked at the IE stage [3]. The strain of HCMV used in those studies was the Towne strain, which has been attenuated by serial passage through human fibroblast cells [3]. Recently, it was discovered that the UL128–131 locus of laboratory strains (Towne and AD169) has mutated because of their repeated passages through fibroblast cells, causing the virus to lose its ability to infect epithelial and endothelial cells [1], [29], [34], [35], [36]. Clinical HCMV strains, or the Ul128–131-repaired AD169 (vDW215-BADrUL131) [29], can infect other types of cells in addition to fibroblast cells, including endothelial cells, lymphocytes, and epithelial cells [29], [37]. We are curious to know whether either the clinical or repaired HCMV can replicate in mouse cells or, alternately, whether it can express more genes in mouse cells than laboratory strains.

**Figure 4:**
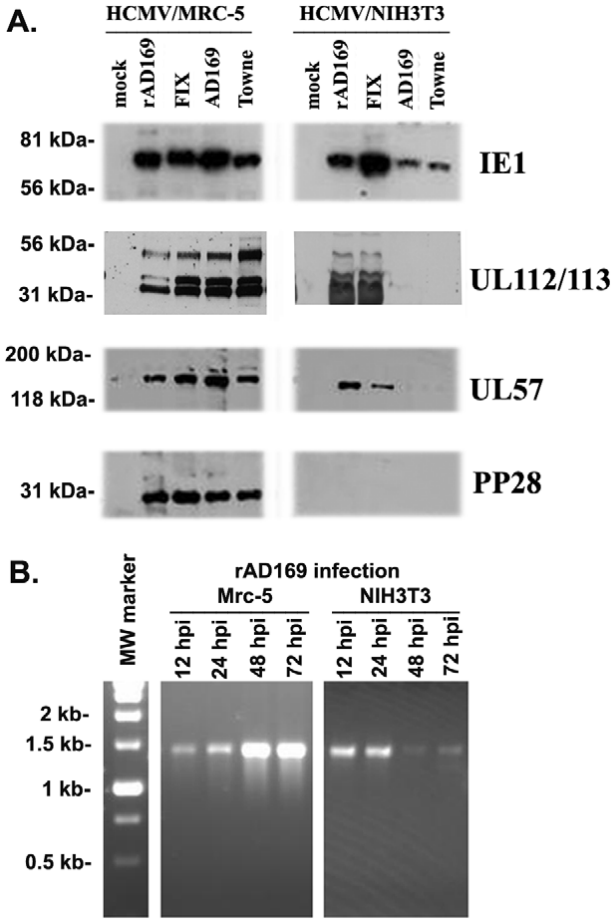
Detection of HCMV proteins and DNA after infection in NIH3T3 and Mrc-5 cells. **A**. Western blot assay to detect HCMV protein production: Whole-cell lysates were prepared at 48 hr after the infection of different strains of HCMV in MRC-5 cells (left) and NIH3T3 cells (right); after being run on PAGE gels, the proteins were transferred and detected with specific antibodies. Detected HMCV proteins are indicated on the right. **B**. PCR to detect HCMV DNA replication: Using a modified Hirt method (7), viral DNA was prepared from MRC-5 cells (left) or NIH3T3 cells (right) at the indicated post-infection times. PCR was performed using the primers (described in the Material and Methods) and PCR products were run on an agarose gel in order to visualize the DNA (using a UV light).

To make this determination, we performed Western blot assays using different antibodies against viral proteins to detect viral-protein production in infected cells. Two laboratory stains (AD169 and Towne), one clinical strain (FIX), and Ul128–131-repaired AD169 (vDW215-BADrUL131) were used to infect either Mrc-5 or NIH3T3 cells for 48 hours, the whole-cell lysates were run on PAGE gels, and Western blot was performed to detect different HCMV proteins, as indicated on the right side of Fig. 4A. As can be seen, the infection of four strains of HCMV can produce all HCMV proteins (IE, E, and late) as detected in Mrc-5 cells; however, infection in NIH3T3 cells was blocked at different stages for different strains. The two laboratory strains (Towne and AD169) of infection were blocked at the IE stage (only IE1 was detectable). Repaired AD169 and the clinical strain (FIX) were blocked at an early stage: several early proteins could be detected, but late protein was not detectable.

To determine whether HCMV could replicate DNA in NIH3T3 cells, we infected either Mrc-5 cells or NIH3T3 cells with repaired AD169; viral DNA was extracted at different times post-infection by the Hirt method [30]. Then, we performed PCR using primers to amplify the DNA of HCMV. If the virus could replicate DNA, the PCR products should show increased amounts of DNA following the infection time, which was the case in the HCMV-infected Mrc-5 cell shown in Fig. 4 B (left); however, the DNA level decreased in the HCMV-infected NIH3T3 cells from 48 hpi. Therefore, HCMV cannot replicate DNA in NIH3T3 cells.

### Distribution of HCMV proteins in NIH3T3 cells

**Figure 5:**
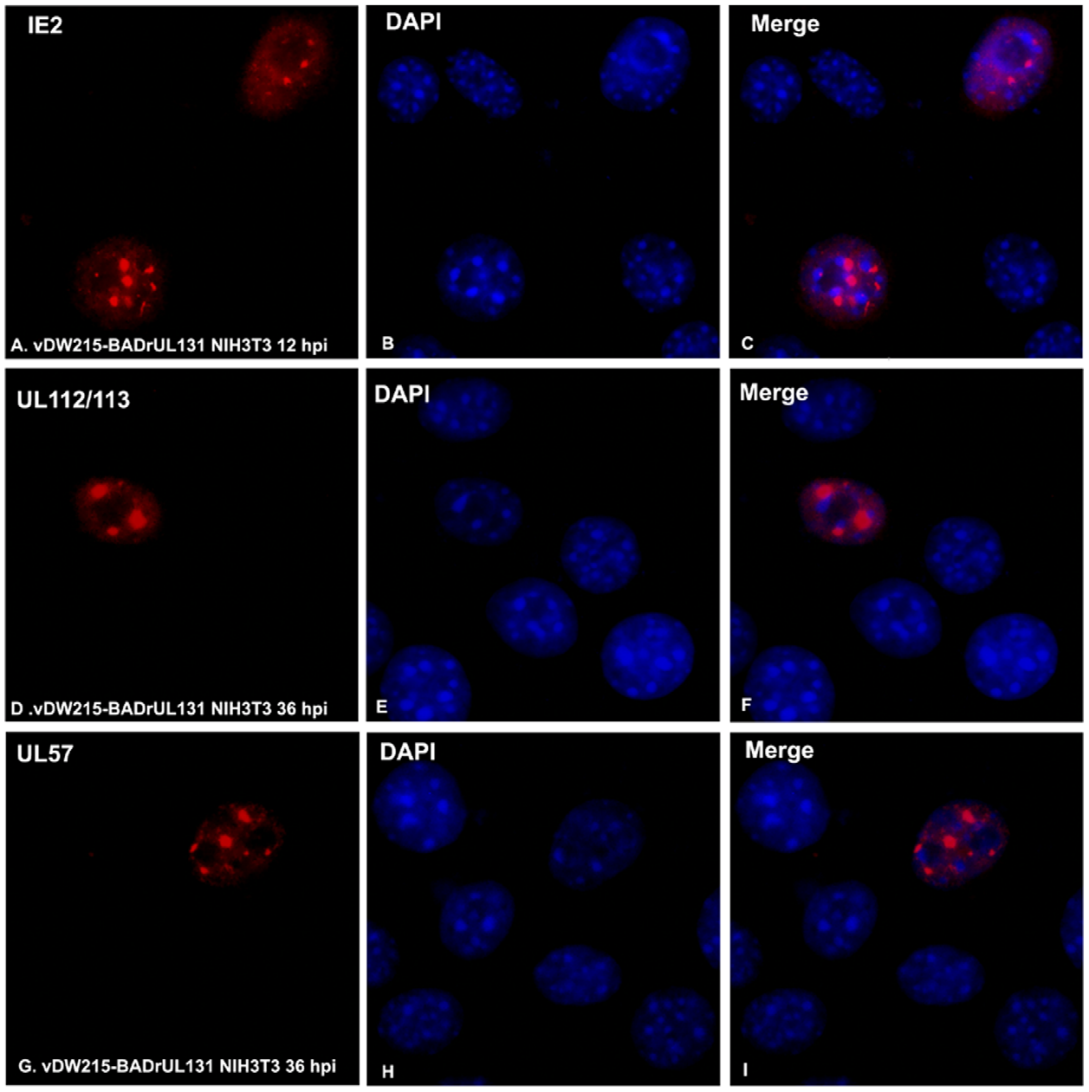
Immunofluorescent assay to detect HCMV proteins after infection in mouse cells. A–C: Detection of HCMV IE2 in red (A), DAPI to show total cells (B), and the two merged in C. D–F: Detection of UL112/113 in red (D), DAPI to show total cells (E), and the two merged in F. G–I: Detection of UL58 in red (G), DAPI to show total cells (H), and the two merged in I.

We observed in the Western blot assay that the repaired HCMV and the clinical strain of HCMV can produce more IE and early (E) gene products; we wondered whether the proteins distribute in these cells as they do in native host cells (human cells). At 12 hpi, we performed IFA to detect IE2 in NIH3T3 cells. As shown in Fig. 5A and C, IE2 distributes in the nucleus as speckles, which is similar to what occurs in native infection. Both UL112/113 (the homology of to the M112/113 of MCMV) and UL 57 were related to viral DNA replication [33], [38] and were produced before DNA replication. In most cases, both proteins form domains in the nucleus before DNA replication in human cells. Here, as can be seen in Fig. 5D and G, the two proteins distributed in NIH3T3 cell as domains. DAPI was used to show the total cells (infected and uninfected). Therefore, the repaired HCMV infection in NIH3T3 cells produced DNA replication-related early proteins, and those proteins distributed in the nucleus as they would in cells infected with the native virus.

### ND10 proteins are strong suppressors of MCMV infection in human cells

**Figure 6:**
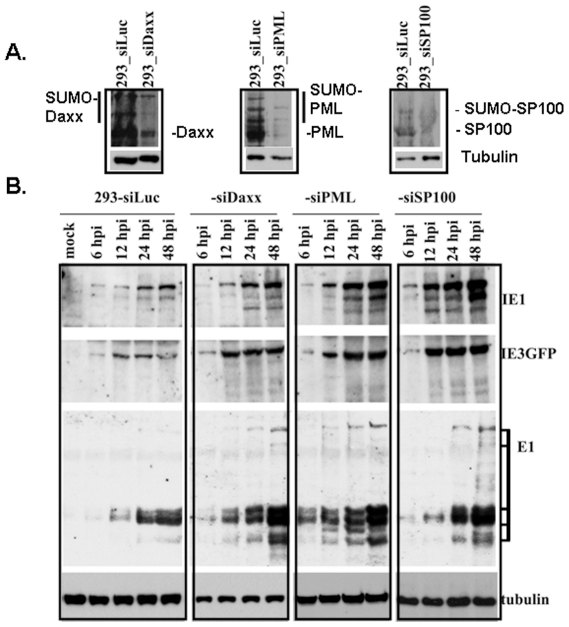
Western blot assay to detect the effects of ND10 proteins (Daxx, PML, and SP100) on MCMV protein production. **A**. siRNA to knockdown the ND10 protein. One hundred pmol of siRNA (the sequences are shown in Materials and Methods) was transfected into HEK293 cells for 24 hours, the whole-cell lysates were run on PAGE gels and probed with antibodies to the targeted proteins, as indicated. Tubulin was used as a house-keeping gene for sample-loading control. siRNA-Luc was used as control. **B**. One hundred pmol of siRNA was used to transfect HEK293 cells in a 24-well plate; 12 hours after transfection, the cells were infected with MCMVIE1/3gfp at an MOI of 1; the cells were harvested at the time indicted, and the whole-cell lysates were used to detect viral-protein production.

The most important ND10 proteins include SP100, Daxx, and PML, all of which have been demonstrated to be intrinsic defense mechanisms against viral infection. To determine whether the ND10 proteins play important roles in preventing MCMV cross-species infection, we used siRNA to knockdown each protein. One hundred pmol of siRNA was transfected into HEK293 cells seeded in 24-well plates; siRNA to Luciferase was used as control. As shown in Fig. 6, 24 hours after the transfection of each siRNA into HEK293 cells, whole-cell lysates were run on PAGE gels, and Western blot assays were performed to detect PML, Daxx, and SP100. The results showed that siRNA effectively repressed gene expression as compared to the loading control protein, tubulin, seen in the bottom of each slide in Fig. 6.

Twenty-four hours after the transfection of 100 pmol of each siRNA into the HEK293 cells, the cells were super-infected with MCMVIE1/3gfp at an MOI of 1; the cells were harvested at different times post-infection, as indicated, and the whole cell-lysates were run on PAGE gels, after which different antibodies were used to ascertain the production of different viral proteins. At this point, we detected IE1, IE3, and early protein 1 (E1). Tubulin was used as sample-loading control. Clearly, after knocking down the ND10 proteins, viral gene products can be increased.

However, knockdown of the ND10 proteins is not enough for MCMV to productively infect human cells because no viral particles can be detected in the human cell; some other mechanism must be involved in blocking the successful infection of MCMV in human cells.

### Knockdown of ND10 proteins enhanced HCMV protein production in mouse cells

**Figure 7:**
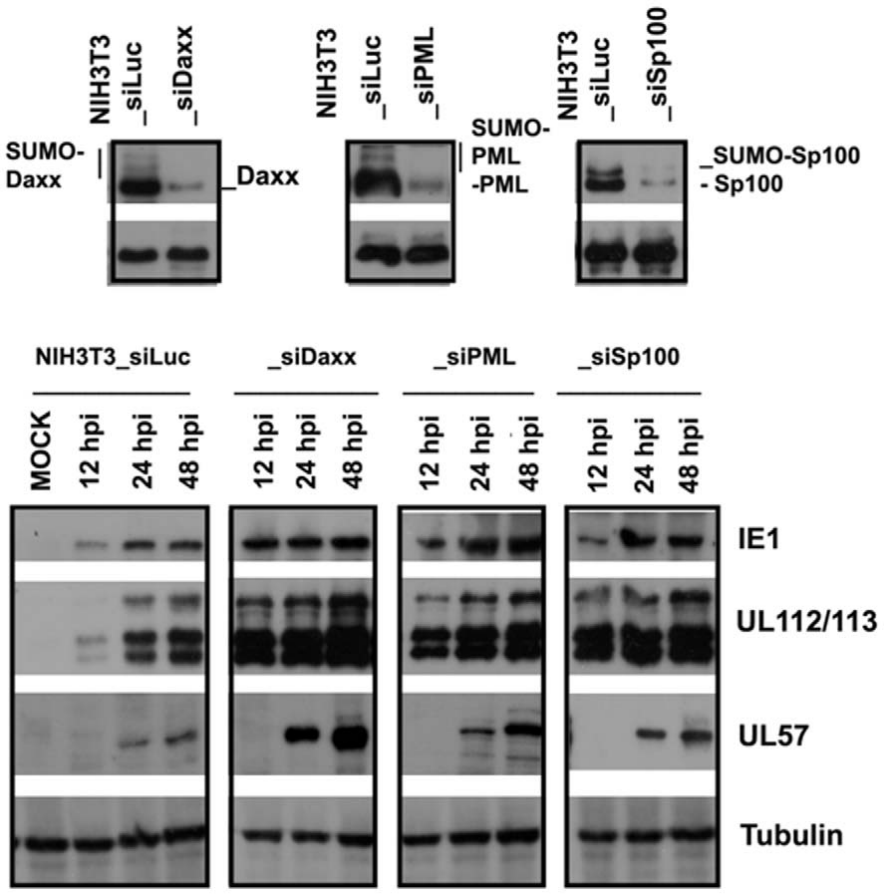
Western blot assay to detect the effects of ND10 proteins (Daxx, PML, and SP100) on HCMV protein production in mouse cells. **A**. siRNA to knockdown the ND10 protein. One hundred pmol of siRNA (the sequences are shown in Materials and Methods) was transfected into NIH3T3 cells for 24 hours, the whole-cell lysates were run on PAGE gels and probed with antibodies to the targeted proteins, as indicated. Tubulin was used as a house-keeping gene for sample-loading control. siRNA-Luc was used as control. **B**. One hundred pmol of siRNA was used to transfect NIH3T3 cells in a 24-well plate; 12 hours after transfection, the cells were infected with repaired AD169 at an MOI of 1, the cells were harvested at the time indicted, and the whole-cell lysates were used to detect viral-protein production.

Using siRNA, we also knocked down ND10 proteins (SP100, Daxx, and PML) from NIH3T3 cells. As shown in Fig. 7, 24 hours after the transfection of each siRNA into NIH3T3 cells, whole-cell lysates were run on PAGE gels, and Western blot assays were performed to detect PML, Daxx, and SP100. The results showed that siRNA effectively repressed gene expression as compared to the loading control protein, tubulin, seen in the bottom of each picture of Fig. 7.

**Figure 8:**
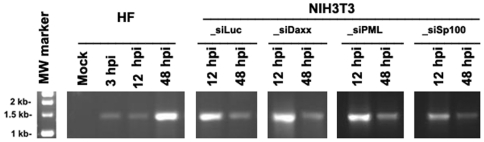
Detection of HCMV DNA after infection in NIH3T3 and Mrc-5 cells. siRNA was used to transfect NIH3T3 cells; 12 hours after transfection, the cells were infected with repaired AD169 at an MOI of 1; using a modified Hirt method (7), viral DNA was prepared from MRC-5 cells (left) or NIH3T3 cells (right) at the indicated post-infection times. PCR was performed using the primers (described in the Material and Methods) and PCR products were run on an agarose gel to visualize the DNA (using a UV light).

Twenty-four hours after the transfection of 100 pmol of each siRNA into NIH3T3 cells, the cells were super-infected with the repaired AD169 at an MOI of 1; the cells were harvested at different times post-infection, as indicated, and the whole cell-lysates were run on PAGE gels, after which different antibodies were used to ascertain the production of different viral proteins. At this point, we detected IE1 and early proteins UL112/113 and UL57. Tubulin was used as sample-loading control. Clearly, after knocking down the ND10 proteins, viral gene products can be increased. However, the essential gene product, pp28 (also called true late protein), was not detectable in all knockdown cells (data not shown). We wondered whether viral DNA replication can be initiated after knocking down the inhibitory genes. Using PCR, we also examined DNA replication in NIH3T3 cells transfected with siRNA against the respective ND10 components; the results showed there to be no increase of DNA in those cells following the infection time course (Fig. 8).

Therefore, ND10 components are important suppressors of viral gene expression, but the knockdown of ND10 proteins cannot lead to a productive CMV cross-species infection. There must be other factors involved in the blocking of HCMV cross-species infection.

## Discussion

In recent years, nuclear domain 10 (ND10), also called PML bodies, has been a topic of intense interest, especially in terms of its role in viral infection [13], [25]. Although a great deal of evidence supports the theory that ND10 components such as PML, Daxx, and SP100 are viral gene repressors and protect host cells against many viruses [14], [16], [21], [22], [26], [39], [40], [41], [42], the effects of the ND10 structure on viral infection have been not determined. The fact that several herpesviruses are able to disrupt ND10 at a very early stage of infection implies that ND10 has a defensive role in the process [9], [10], [11], [26]. However, several DNA viruses (such as herpesviruses) dock their input DNA, replicate DNA, and transcribe immediate-early genes at ND10, which argues that ND10 favors viral replication [10]. To comparatively investigate the roles of ND10 and ND10 proteins in cross-species infections (MCMV in human cells and HCMV in mouse cells), we performed an immunofluorescence assay to detect the effects of CMV infection on ND10. We discovered that during the infection of MCMV in human cells, MCMV IE1 distributed both diffusely and as domains (Fig. 2), which differs from what has been found in mouse cells, where IE1 distributes only diffusely. In addition, MCMV IE1 loses the ability to disrupt the ND10 of human cells. HCMV IE1 also loses the ability to disperse mouse cell ND10.

Previously, it was found that laboratory strains of HCMV infection in mouse cells can express only IE1 and not IE2, even though that IE1 shares a promoter and its first three exons with IE2. Therefore, it was concluded that HCMV infection in mouse cells was blocked at the IE stage [3]. However, it has been reported that the laboratory strains of HCMV experience profound mutations during replication in human fibroblast cells [29], [34], [35], [36], [43]. The mutations lead not only to the attenuation of HCMV but also to the narrowing of cell tropisms. The mutation of HCMV cell tropism occurred at gene UL128–131 since the repaired UL128–131 caused the recovery of cell tropisms [29], [34], [35], [36], [43]. Therefore, this time using both the clinical HCMV strain and the repaired HCMV, we reentered the study of HCMV infection in mouse cells. At this point, we found that HCMV could produce IE proteins and some early proteins (Fig. 4 and 5), but failed to replicate DNA. We now conclude that HCMV infection in mouse cells was blocked before DNA replication.

Unlike what takes place in RNA viruses (in which species specificity is determined by the interaction of viral proteins and cellular receptors) [44], the species-specific restriction of CMV occurs not at the entry to cells but at the post-DNA replication stage for MCMV infection in human cells [3], [45], [46], [47] and at the early stage before DNA replication for HCMV infection in mouse cells. Cellular proteins, including transcription repressors (Daxx, PML, SP100), have suppressive effects on viral gene expression and represent an intrinsic, host-cell defense [26], [41], [48], [49]. PML is the scaffold protein and is essential for the formation of ND10 because PML knockout (k/o) cells lack ND10, and inducing exogenous PML into PML knockout cells can restore ND10 [50]. SUMOylation is another characteristic of PML and makes it possible for PML to interact with many other nuclear proteins. There are more than 70 different cellular proteins that have been found to be related to ND10, and the proteins that interact with PML have already been reviewed by Dr. Van Ostade1 and colleagues [51]. The most frequently investigated PML-interacting proteins include Daxx and SP100. ND10 structure can be shown by indirect immunofluorescence using anti-PML, -Daxx, and -SP100 antibodies.

The inhibitory effects of ND10 proteins on viral infection have been demonstrated on PML, Daxx, and SP100. The effects of the ND10 structure on viral infection have not been determined. ND10's defensive role in the infection process can be inferred by the fact that several herpesviruses are required to disrupt it. We previously showed that IE1 is the only protein of MCMV that is capable of disrupting the ND10 of mouse cells [26]. In that prior study, we reported that the IE1 of MCMV also colocalized with the ND10 of human cells; however, IE1 lost its ability to disperse ND10 in cytomegalovirus cross-species infections. This discovery supports the theory that ND10 might block the productive cross-species infection of cytomegalovirus. Consistent with this speculation, we found that human-cell ND10 proteins, such as SP100, PML, and Daxx, strongly suppress MCMV viral gene expression (Fig. 6), and mouse cell ND10 protein also represses HCMV gene expression (Fig. 7).

Interestingly, HCMV laboratory-strain infections in mouse cells can produce IE1 but not IE2, even though IE1 and IE2 share a promoter and the first three exons, all of which suggests that splicing regulation also plays a role in blocking HCMV infection in mouse cells.

In summary, we discovered that intrinsic cellular defense mechanisms participate in the blocking of CMV cross-species infection and that CMV IE1 loses its ability to disperse ND10. In HCMV-infected mouse cells, only IE1 (and not IE2) can be detected in laboratory-strain–infected mouse cells, but clinical strains and UL128–131-repaired strains can produce many more viral gene products. Future studies will focus on identifying the additional mechanisms that are involved in blocking cross-species infection.

## References

[1] Sinzger C, Digel M, Jahn G (2008). Cytomegalovirus cell tropism.. Curr Top Microbiol Immunol.

[2] Weller TH (1970). Review. Cytomegaloviruses: the difficult years.. J Infect Dis.

[3] Lafemina RL, Hayward GS (1988). Differences in cell-type-specific blocks to immediate early gene expression and DNA replication of human, simian and murine cytomegalovirus.. J Gen Virol.

[4] Smith CB, Wei LS, Griffiths M (1986). Mouse cytomegalovirus is infectious for rats and alters lymphocyte subsets and spleen cell proliferation.. Arch Virol.

[5] Bruggeman CA, Meijer H, Dormans PH, Debie WM, Grauls GE (1982). Isolation of a cytomegalovirus-like agent from wild rats.. Arch Virol.

[6] Mocarski ES, Shenk T, Pass R F,  Knipe D M,  Howley P M (2006). Cytomegaloviruses;.

[7] Tang Q, Maul GG (2006). Mouse cytomegalovirus crosses the species barrier with help from a few human cytomegalovirus proteins.. J Virol.

[8] Jurak I, Brune W (2006). Induction of apoptosis limits cytomegalovirus cross-species infection.. EMBO J.

[9] Ahn JH, Brignole EJ, Hayward GS (1998). Disruption of PML subnuclear domains by the acidic IE1 protein of human cytomegalovirus is mediated through interaction with PML and may modulate a RING finger-dependent cryptic transactivator function of PML.. Mol Cell Biol.

[10] Ishov AM, Stenberg RM, Maul GG (1997). Human cytomegalovirus immediate early interaction with host nuclear structures: definition of an immediate transcript environment.. J Cell Biol.

[11] Everett RD, Maul GG (1994). HSV-1 IE protein Vmw110 causes redistribution of PML.. Embo J.

[12] Saffert RT, Kalejta RF (2008). Promyelocytic leukemia-nuclear body proteins: herpesvirus enemies, accomplices, or both?. Future Virol.

[13] Tavalai N, Stamminger T (2008). New insights into the role of the subnuclear structure ND10 for viral infection.. Biochim Biophys Acta.

[14] Tavalai N, Papior P, Rechter S, Leis M, Stamminger T (2006). Evidence for a role of the cellular ND10 protein PML in mediating intrinsic immunity against human cytomegalovirus infections.. J Virol.

[15] Roberts S, Hillman ML, Knight GL, Gallimore PH (2003). The ND10 component promyelocytic leukemia protein relocates to human papillomavirus type 1 E4 intranuclear inclusion bodies in cultured keratinocytes and in warts.. J Virol.

[16] Negorev DG, Vladimirova OV, Maul GG (2009). Differential functions of interferon-upregulated Sp100 isoforms: herpes simplex virus type 1 promoter-based immediate-early gene suppression and PML protection from ICP0-mediated degradation.. J Virol.

[17] Ling PD, Peng RS, Nakajima A, Yu JH, Tan J (2005). Mediation of Epstein-Barr virus EBNA-LP transcriptional coactivation by Sp100.. EMBO J.

[18] Gu H, Roizman B (2003). The degradation of promyelocytic leukemia and Sp100 proteins by herpes simplex virus 1 is mediated by the ubiquitin-conjugating enzyme UbcH5a.. Proc Natl Acad Sci U S A.

[19] Puvion-Dutilleul F, Venturini L, Guillemin MC, de The H, Puvion E (1995). Sequestration of PML and Sp100 proteins in an intranuclear viral structure during herpes simplex virus type 1 infection.. Exp Cell Res.

[20] Chelbi-Alix MK, de The H (1999). Herpes virus induced proteasome-dependent degradation of the nuclear bodies-associated PML and Sp100 proteins.. Oncogene.

[21] Saffert RT, Kalejta RF (2007). Human cytomegalovirus gene expression is silenced by Daxx-mediated intrinsic immune defense in model latent infections established in vitro.. J Virol.

[22] Woodhall DL, Groves IJ, Reeves MB, Wilkinson G, Sinclair JH (2006). Human Daxx-mediated repression of human cytomegalovirus gene expression correlates with a repressive chromatin structure around the major immediate early promoter.. J Biol Chem.

[23] Saffert RT, Kalejta RF (2006). Inactivating a cellular intrinsic immune defense mediated by Daxx is the mechanism through which the human cytomegalovirus pp71 protein stimulates viral immediate-early gene expression.. J Virol.

[24] Hofmann H, Sindre H, Stamminger T (2002). Functional interaction between the pp71 protein of human cytomegalovirus and the PML-interacting protein human Daxx.. J Virol.

[25] Maul GG (2008). Initiation of cytomegalovirus infection at ND10.. Curr Top Microbiol Immunol.

[26] Tang Q, Maul GG (2003). Mouse cytomegalovirus immediate-early protein 1 binds with host cell repressors to relieve suppressive effects on viral transcription and replication during lytic infection.. J Virol.

[27] Schumacher U, Handke W, Jurak I, Brune W (2010). Mutations in the M112/M113-coding region facilitate murine cytomegalovirus replication in human cells.. J Virol.

[28] Grazia Revello M, Baldanti F, Percivalle E, Sarasini A, De-Giuli L (2001). In vitro selection of human cytomegalovirus variants unable to transfer virus and virus products from infected cells to polymorphonuclear leukocytes and to grow in endothelial cells.. J Gen Virol.

[29] Wang W, Taylor SL, Leisenfelder SA, Morton R, Moffat JF (2005). Human cytomegalovirus genes in the 15-kilobase region are required for viral replication in implanted human tissues in SCID mice.. J Virol.

[30] Eizuru Y, Inagawa S, Minamishima Y (1984). Application of “Hirt supernatant” DNA to the molecular epidemiology of cytomegalovirus infections.. J Clin Microbiol.

[31] Tang Q, Bell P, Tegtmeyer P, Maul GG (2000). Replication but not transcription of simian virus 40 DNA is dependent on nuclear domain 10.. J Virol.

[32] Everett RD (2006). Interactions between DNA viruses, ND10 and the DNA damage response.. Cell Microbiol.

[33] Martinez FP, Cosme RS, Tang Q (2010). Murine cytomegalovirus major immediate-early protein 3 interacts with cellular and viral proteins in viral DNA replication compartments and is important for early gene activation.. J Gen Virol.

[34] Hahn G, Revello MG, Patrone M, Percivalle E, Campanini G (2004). Human cytomegalovirus UL131–128 genes are indispensable for virus growth in endothelial cells and virus transfer to leukocytes.. J Virol.

[35] MacCormac LP, Grundy JE (1999). Two clinical isolates and the Toledo strain of cytomegalovirus contain endothelial cell tropic variants that are not present in the AD169, Towne, or Davis strains.. J Med Virol.

[36] Ryckman BJ, Rainish BL, Chase MC, Borton JA, Nelson JA (2008). Characterization of the human cytomegalovirus gH/gL/UL128–131 complex that mediates entry into epithelial and endothelial cells.. J Virol.

[37] Cheng B, Martinez FP, Katano H, Tang Q (2009). Evidence of inability of human cytomegalovirus to reactivate Kaposi's sarcoma-associated herpesvirus from latency in body cavity-based lymphocytes.. J Clin Virol.

[38] Ahn JH, Jang WJ, Hayward GS (1999). The human cytomegalovirus IE2 and UL112–113 proteins accumulate in viral DNA replication compartments that initiate from the periphery of promyelocytic leukemia protein-associated nuclear bodies (PODs or ND10).. J Virol.

[39] Everett RD, Chelbi-Alix MK (2007). PML and PML nuclear bodies: implications in antiviral defence.. Biochimie.

[40] Preston CM, Nicholl MJ (2006). Role of the cellular protein hDaxx in human cytomegalovirus immediate-early gene expression.. J Gen Virol.

[41] Cantrell SR, Bresnahan WA (2005). Interaction between the human cytomegalovirus UL82 gene product (pp71) and hDaxx regulates immediate-early gene expression and viral replication.. J Virol.

[42] Jiang M, Entezami P, Gamez M, Stamminger T, Imperiale MJ (2011). Functional Reorganization of Promyelocytic Leukemia Nuclear Bodies during BK Virus Infection.. MBio.

[43] Wang D, Shenk T (2005). Human cytomegalovirus virion protein complex required for epithelial and endothelial cell tropism.. Proc Natl Acad Sci U S A.

[44] Louz D, Bergmans HE, Loos BP, Hoeben RC (2005). Cross-species transfer of viruses: implications for the use of viral vectors in biomedical research, gene therapy and as live-virus vaccines.. J Gene Med.

[45] Walker DG, Hudson JB (1988). Further characterization of the murine cytomegalovirus induced early proteins in permissive and nonpermissive cells.. Arch Virol.

[46] Walker D, Hudson J (1987). Analysis of immediate-early and early proteins of murine cytomegalovirus in permissive and nonpermissive cells.. Arch Virol.

[47] Misra V, Muller MT, Hudson JB (1977). The enumeration of viral genomes in murine cytomegalovirus-infected cells.. Virology.

[48] Kalejta RF, Shenk T (2003). The human cytomegalovirus UL82 gene product (pp71) accelerates progression through the G1 phase of the cell cycle.. J Virol.

[49] Ishov AM, Vladimirova OV, Maul GG (2002). Daxx-mediated accumulation of human cytomegalovirus tegument protein pp71 at ND10 facilitates initiation of viral infection at these nuclear domains.. J Virol.

[50] Ishov AM, Sotnikov AG, Negorev D, Vladimirova OV, Neff N (1999). PML is critical for ND10 formation and recruits the PML-interacting protein daxx to this nuclear structure when modified by SUMO-1.. J Cell Biol.

[51] Van Damme E, Laukens K, Dang TH, Van Ostade X (2010). A manually curated network of the PML nuclear body interactome reveals an important role for PML-NBs in SUMOylation dynamics.. Int J Biol Sci.

